# Fat quantification in the sacroiliac joint syndesmosis: a new semi-automatic volumetric approach

**DOI:** 10.1038/s41598-023-44066-x

**Published:** 2023-10-07

**Authors:** Amélie Poilliot, Louis Zeissloff, Benjamin Ondruschka, Niels Hammer

**Affiliations:** 1 Anatomical Institute, University of Basel, Pestalozzistrasse 20, 4056 Basel, Switzerland; 2 CLEARSY, Safety Solutions Designer, Strasbourg, France; 3 Institute of Legal Medicine, University Medical Center Hamburg-Eppendorf, Hamburg, Germany; 4 Division of Macroscopic and Clinical Anatomy, Gottfried Schatz Research Center, Medical University of Graz, Auenbruggerplatz 25, Graz, Austria; 5 University Clinics, University of Leipzig, Leipzig, Germany; 6 Division of Biomechatronics, Fraunhofer Institute for Machine Tools and Forming Technology (IWU), Dresden, Germany

**Keywords:** Imaging, Software, Structure determination, Biological techniques, Anatomy, Medical research

## Abstract

Fat is physiologically embedded within the interosseous ligaments in the posterior part of the sacroiliac joint (PSIJ). This composite of fat and ligaments is hypothesized to serve a shock-absorbing, stabilizing function for the sacroiliac joint and the lumbopelvic transition region. Using a novel Python-based software (VolSEQ), total PSIJ volume and fat volume were computed semi-automatically. Differences within the cohort and the viability of the program for the quantification of fat in routine computed tomography (CT) scans were assessed. In 37 CT scans of heathy individuals, the PSIJ were first manually segmented as a region of interest in OSIRIX. Within VolSEQ, ‘fat’ Hounsfield units (− 150 to − 50 HU) are selected and the DICOM file of the patient scan and associated region of interest file from OSIRIX were imported and the pixel sub volumes were then automatically computed. Volume comparisons were made between sexes, sides and ages (≤ 30, 31–64 and > 65 years). PSIJ volumes in both software (VolSeq vs. OSIRIX) were non-different (both 9.7 ± 2.8cm^3^;* p* = 0.9). Total PSIJ volume (*p* = 0.3) and fat volume (*p* = 0.7) between sexes were non-different. A significant difference in total PSIJ volume between sexes (*p* < 0.01) but not in fat volume (*p* = 0.3) was found only in the ≥ 65 years cohort. Fat volume within the PSIJ remains unchanged throughout life. PSIJ volume is sex-dependent after 65 years. VolSEQ is a viable and user-friendly method for sub-volume quantification of tissues in CT.

## Introduction

Quantification of tissue volumes and sub volumes using medical imaging is not a new technique^1–8^. Commonly, data sets derived from magnetic resonance imaging (MRI)^2,9–15^ or computed tomography (CT) are used for this purpose. Available software such as OSIRIX (Pixmeo Sarl 2016, Geneva, Switzerland), Horos (The Horos Project, MD, USA; https://horosproject.org/), SliceOMatic (TomoVision, Montreal, QC, Canada), Amira (Zuse Institute Berlin, Germany) or ImageJ (National Institutes of Health, Bethesda, MA, USA) are well established tools for both the qualitative and quantitative assessment of images based on the manual segmentation of regions of interest (ROI)^1–6,9–15^. The quantification of certain tissues in small specific regions is often challenged by the limited resolution of clinical CT and MRI datasets^16–18^ and has to be performed manually with all consequences of potential subjectivity. Such an approach is more likely to result in inaccurate estimations, even for anatomically experienced evaluators.

**Figure 1:**
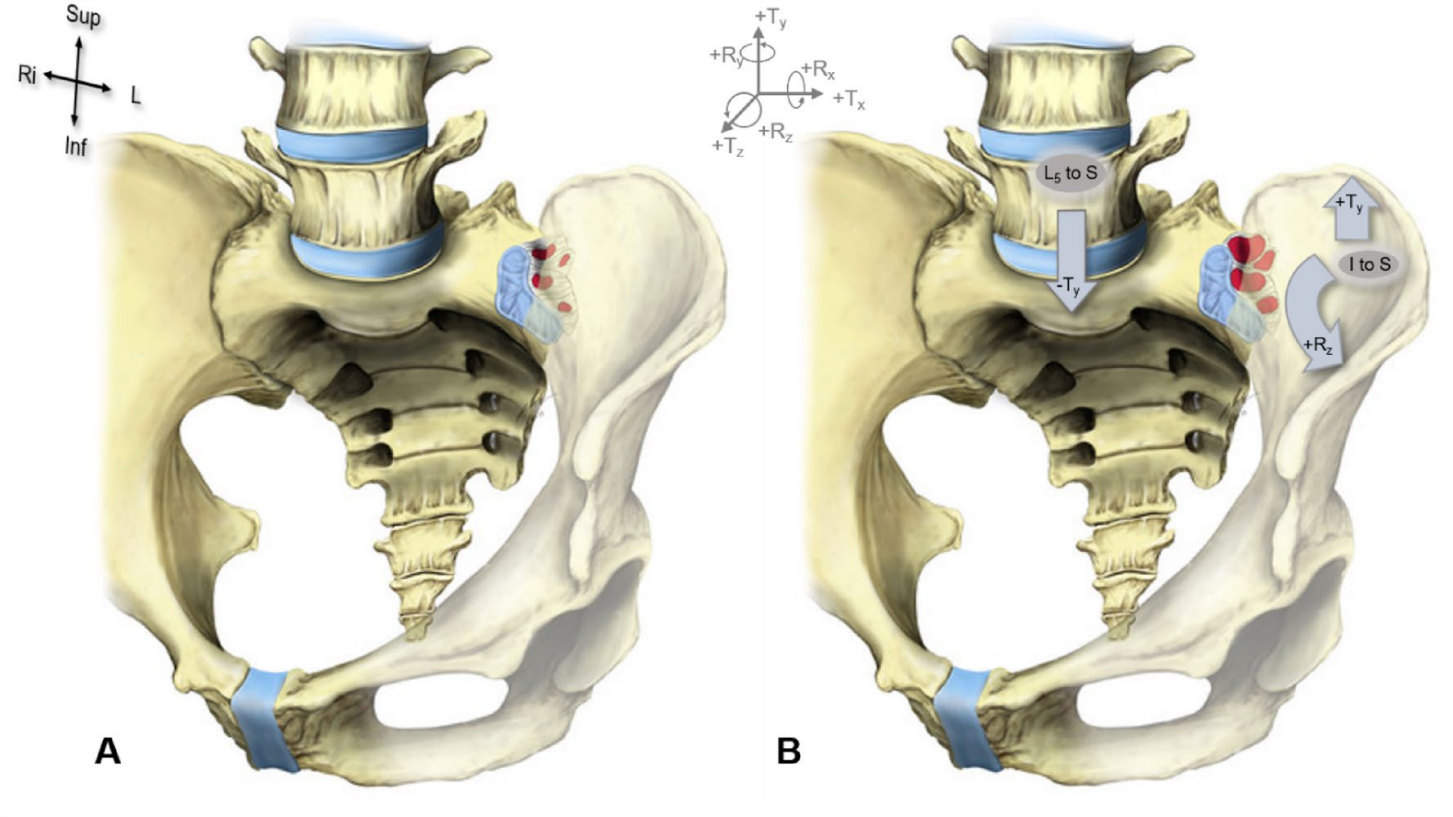
Demonstration of the cushioning phenomenon in the posterior SIJ region in response to the integration kinematics at the SIJ. In red is the fat within the white interosseous ligaments. (**A**) The SIJ under no force, the ligaments are tense (**B**) the SIJ is under force or load compression called ‘integration’ (arrows), the fat can be seen here deforming within the relaxed interosseous ligaments. I: ilium, Inf: inferior, L: left, R: rotation movement, Ri: right, S: sacrum, Sup: superior T: translation movement.

Within the posterior sacroiliac region (PSIJ), fat tissue is physiologically embedded within the meshes of the interosseous and posterior sacroiliac ligaments^7,19,20^, forming a mattress-like configuration (Fig. 1). These deposits have been hypothesized by our group to have a shock-absorbing and stabilizing function for the sacroiliac joint (SIJ) and the lumbopelvic transition region^21^ and serve as protection for the neurovascular structures to travel and supply the syndesmotic SIJ. Structural fat here is thought to partly compensate for the incongruent surfaces of the sacrum and ilium under continuous loading while walking, in single and in double leg stance^7,22^. During compression, tension and shear of the SIJ in the loaded state, adipose tissue enables the effective stress distribution within the ligamentous structures and acts as a ‘cushion’ for the structures within the PSIJ^7^. A previous study^7^ successfully quantified the total fat volume in this region and determined sex and age-related differences in fat content using OSIRIX. Major drawbacks were that it was limited to an elderly population and the segmentation was based on digital photographs of cadaveric specimens, which was a time-consuming and potentially subjective approach as some smaller fat pixels may be invisible to the naked eye. Thus, a more recent study by Poilliot et al.^8^ introduced a semi-automated method using MATLAB (MathWorks, Natick, MA, USA) to quantify fat within the PSIJ using clinical CT scans. Although fat is often visualized using MRI, the small volumes of tissue in the PSIJ requires the methodology to be more specific in detecting volumes to a pixel level. Therefore, CT imaging was selected over MRI as it allows for the objective quantification of pixels based on HU values which are representative of various tissues based on a known Hounsfield scale^23^. This method was found to be objective and time efficient with a higher degree of sensitivity than the previous manual segmentation method in Poilliot et al.^7^.

A major drawback of the semi-automated method utilized in Poilliot et al.^8^ was that the study was limited to a small number of CT scans and these were all elderly individuals. Furthermore, utilizing MATLAB requires training and may therefore be unaccessible to certain individuals, not to mention costly. Therefore, using a similar approach, a novel cost-effective Python program (VolSEQ) was created to quantify the fat content in the PSIJ region in a cohort of healthy individuals using CT scans with a larger age range in adulthood. This given research work aims to determine the total volume of the PSIJ and subsequently compute the fat volume in the region to determine differences within a generic ‘healthy’ SIJ population and test the viability of the VolSEQ program for the quantification of fat in CT scans.

The following hypotheses were investigated and revisited using this new software:

- Fat volume decreases with age within the sacroiliac joint
- Females have a higher fat content as well as total PSIJ volumes than males
- Side has no influence on fat content

## Materials and methods

### Patients

A total of 37 routine clinical CT scans (19 females, 18 males; age range 16–82 years) were used in this study. Slice thickness was 1.25 mm with a recon increment of 1 mm. None of the included patients had a known history of lower back pain, SIJ-related pathology nor abnormalities on previous medical records.

Ethical approval was acquired for the use of patient datasets used in research studies for diagnostic and therapeutic purposes as in other works^24,25^. These patients were used in previous studies for the completion of a student thesis by the author^24,25^. Approval was granted on the grounds of existing patient datasets. Informed consent was obtained from all living subjects of this study. All methods were carried out in accordance with relevant guidelines and regulations. The University of Otago Human Ethics Committee (Health) [ref: H17/20]) approved the present study. All the data were analyzed strictly anonymously.

### Fat quantification using VolSEQ

**Figure 2:**
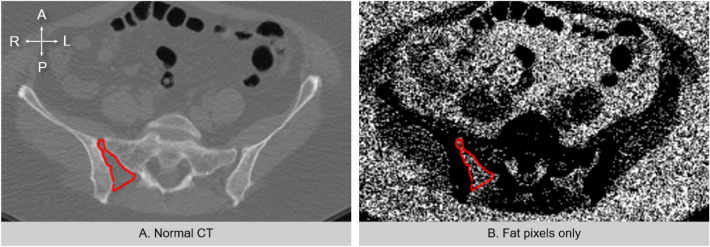
Computed tomography (CT) results when applying the Hounsfield unit range in VolSEQ (− 150 to − 50 HU), axial view. (**A**) Normal CT with the posterior sacroiliac region (PSIJ) highlighted in red. The posterior boundary is an arbitrary line running between ilium and sacrum (**B**) The same slice with only pixels of fat (in white). A: anterior, P: posterior, L: left, R: right.

Using a Python algorithm, a novel software (VolSEQ v1.0.0, Python, freely available at: https://github.com/4lom/VolSEQ.git) was developed by software developer Zeissloff^26^ for the purpose of this study. VolSEQ is intended to help quantify pixels of selected tissue based on a known range of Hounsfield units (HU) on conventional CT scans (SOMATOM as64 open, Siemens, Munich, Germany; Aquilion one, Toshiba, Tokyo, Japan). Imaging was performed in the axial plane using a 1.25 mm slice thickness, and 500-mm field-of-view. Image acquisition parameters included: voltage of 120 kV, X-ray tube current 40–160 mA with an exposure time of 505 ms. In this study, only HUs representing fat in the CT scans were targeted based on the range − 150 to − 50 HU, as defined in previous studies^1,4,8^. The regions of interest (ROIs) were applied to the patient series when it was processed using VolSEQ to act as a boundary to only quantify pixels in the PSIJ area of the image using pre-made ROIs. The ROIs were first manually outlined in OSIRIX on each slice, and the specific ROI information was exported into a converted ‘.csv’ file usable in Excel (Microsoft Corp., Redmont, WA, USA). The boundaries of the PSIJ have been described previously^7,8^. Since the boundaries of the posterior sacroiliac ligament were difficult to clearly visualize in the CT scans, the posterior boundary was set as an arbitrary line running directly between the ilium and the most posterior part of the sacrum (Fig. 2A). Results computed by the program were the pixelated volume of fat within the PSIJ converted into a volume in cubic centimeters (Fig. 2B). Furthermore, total volume was computed in OSIRIX and in VolSEQ to compare the reliability of the calculations of the program.

### Subgroup analyses

**Figure 3:**
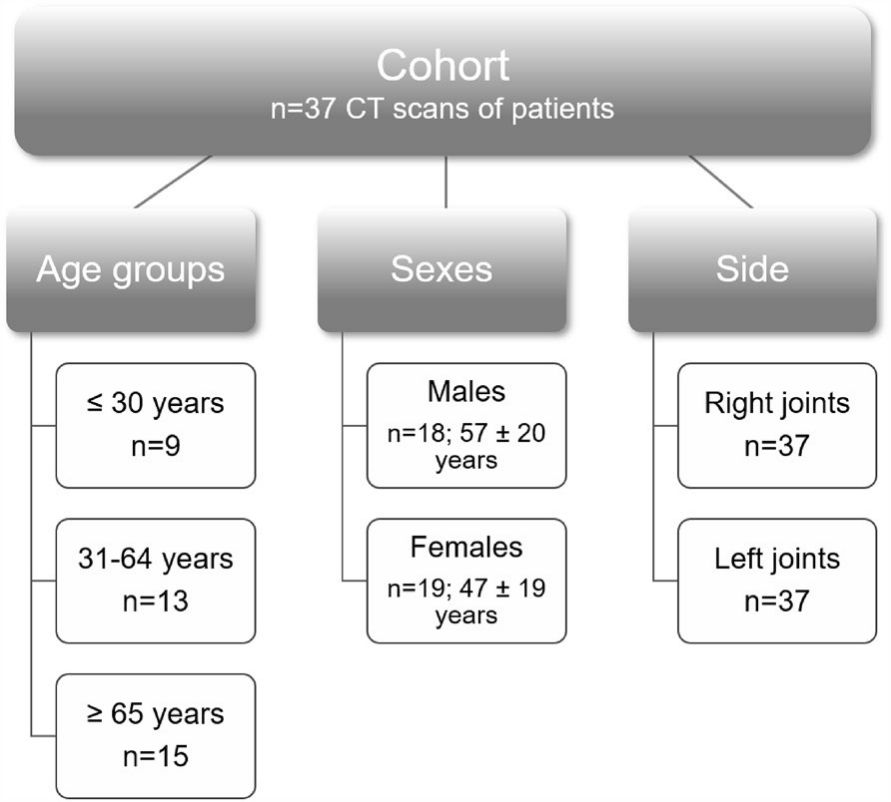
Schematic representation of the cohort and subgroups used for the study. Please note that none of these cases had a current or past history of low back pain, sacroiliac joint-related pathology or abnormalities on previous medical records and visible sacroiliac joint conditions on the CT (computed tomography) scans.

For the purpose of analyzing and describing fat content in the PSIJ region in the given cohort, various subgroups were created for comparison purposes. For age group comparisons, three different age groups were created: young ≤ 30 years, middle-aged 31–64 years and older ≥ 65 years groups. Moreover, the cohort was also subdivided by sex and side (Fig. 3).

### Statistical analyses

Prism (version 7.0a, GraphPad, San Diego, CA, USA) was used for statistical analyses. Normal distribution of the data was determined using the Shapiro–Wilk test. To assess differences between total volumes using OSIRIX and VolSEQ, a non-parametric Mann–Whitney U test was subsequently used as the data were not normally distributed as well as a Bland–Altman plot. For fat volume comparisons between sexes and sides and fat volume comparisons between age groups, an ANOVA test or Kruskal–Wallis test was employed depending on the distribution of the data. Age correlations were assessed using a two-tailed Spearman-r test as data were non-parametric. A perfect correlation was defined as equal to 1.0, very strong as ≥ 0.8, moderate as ≥ 0.6, fair as ≥ 0.3, and weak ≥ 0.1 based on Chan^27^. *P* values of 0.05 or less were considered statistically significant. Values are given as means ± standard deviations.

## Results

### OSIRIX and VoISEQ yield similar accuracy and mean values for PSIJ volumes

**Figure 4:**
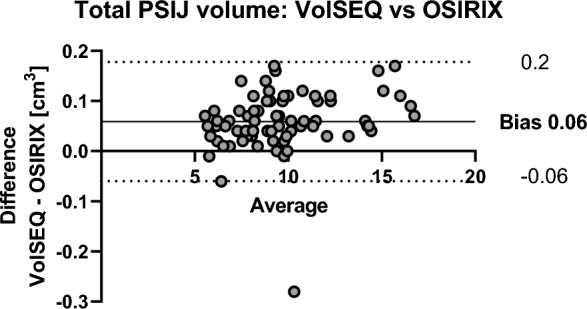
Bland–Altman plot comparing difference in volume assessment on the posterior part of the sacroiliac joint (PSIJ) region using OSIRIX and VolSEQ.

The Bland–Altman plot revealed a small bias when comparing the total volumes of the PSIJ between both methods (0.06 cm^3^) with narrow limits of agreement (− 0.06 and 0.2 cm^3^; Fig. 4). No significant difference (*p* = 0.9) was found between the total PSIJ volumes using OSIRIX (mean 9.7 ± 2.8 cm^3^, 95% CI 9.1–10.4 cm^3^) and VolSEQ (mean 9.7 ± 2.8 cm^3^, 95% CI 9.0–10.3 cm^3^).

### Both mean fat and total volume of the PSIJ syndesmosis were similar across the three age spans ≤ 30 to ≥ 65 years using VolSEQ

**Figure 5:**
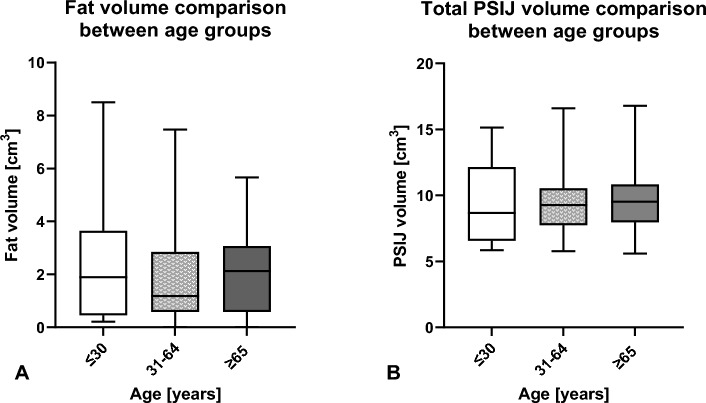
Fat volume (**A**) and total volume of the posterior part of the sacroiliac joint (PSIJ), (**B**) between three age groups (≤ 30 years, 31–64 years, and ≥ 65 years) using VolSEQ. The outlines of the boxes indicate the 25% and 75% percentile, the solid horizontal black line the median. The whiskers indicate the minima and maxima.

Variation in fat volume seems to decrease with age (Fig. 5A). No significant difference was observed in both fat volume and total PSIJ volume between all three age groups grouped (Fig. 5).

### Though total PSIJ volume was non-different between sexes, fat volume differed between males and females aged beyond 65 years

No difference was found when comparing total PSIJ volume (*p* = 0.3) and fat volume (*p* = 0.7) between all males and females. The total PSIJ volume in females was: mean 10.3 ± 2.9 cm^3^ (95% CI 9.4–11.3 cm^3^); in males: mean 9.1 ± 2.6 cm^3^ (95% CI 8.2–10.0 cm^3^). Fat volume in females was: mean 2.4 ± 2.1 cm^3^ (95% CI 1.7–3.1 cm^3^); in males: mean 1.9 ± 1.8 cm^3^ (95% CI 1.3–2.5 cm^3^). Nor was there any significance between the age groups when comparing a single sex for both sexes (*p* > 0.9).

**Figure 6:**
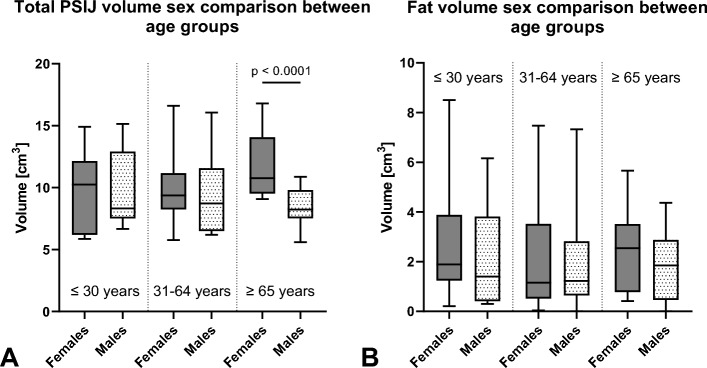
Sex comparison of (**A**) total volume of the posterior part of the sacroiliac joint (PSIJ) volume and (**B**) fat volume between the younger age group (≤ 30 years), middle age (31–64) and the older age group (≥ 65 years). The outlines of the boxes indicate the 25% and 75% percentile, the solid horizontal black line the median. The whiskers indicate the minima and maxima.

Further un-pooled analyses of aged individuals (≥ 65 years) revealed a significant difference in total PSIJ volume between sexes (*p* < 0.01) (Fig. 6A), but not in fat volume (*p* = 0.3) (Fig. 6B). When looking at younger (≤ 30 years) and middle age (31–64 years) groups, neither the total PSIJ volume nor the fat volume were significantly different (all* p* > 0.9) (Fig. 6).

### No difference was found in total volume between sides, however, fat percentage was higher on the right side

**Figure 7:**
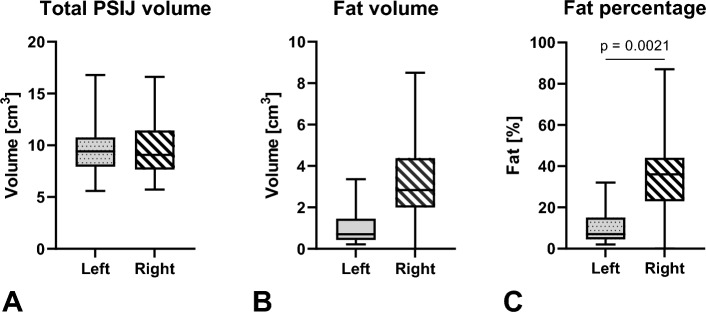
Left and right side PSIJ (posterior part of the sacroiliac joint) comparisons of (**A**) total volume and (**B**) fat volume and (**C**) the fat percentage (calculated as fat volume/Total PSIJ volume × 100). The outlines of the boxes indicate the 25% and 75% percentile, the solid horizontal black line the median. The whiskers indicate the minima and maxima.

No significant difference was found between left and right sides when comparing the total PSIJ volume (*p* > 0.9) and fat volumes (*p* = 0.1) (Fig. 7A,B). However, a significant difference was found between fat percentage between the left and right sides (*p* = 0.01) (Fig. 7C).

### PSIJ fat volume appeared not to correlate with age but it does moderately correlate with fat volume

**Figure 8:**
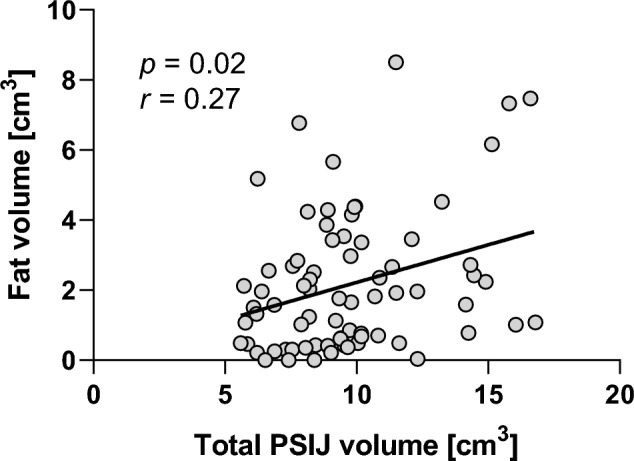
Linear correlation between fat volume and total volume of the posterior part of the sacroiliac joint (PSIJ).

No significant correlation was found between age and fat volume or fat percentage (*p* = 0.9), and significance remained unchanged when further separated by sex (*p* > 0.7). There was however a weak to fair positive linear correlation (*r* = 0.27; *p* = 0.02) between fat volume and total PSIJ volume (Fig. 8).

## Discussion

This paper offers first results using a novel semi-automated method to quantify tissue volumes, allowing for a time-effective approach applicable to the posterior sacroiliac joint region. This can be applied to research on the impact of certain tissue volumes (like fat) that may the function or the potential development of a certain pathology at a specific location: e.g., sarcopenia, without needing to use cadaveric material. In the here given application, adipose tissue was targeted in defined regions of interest with HU between − 150 and − 50 on regular CT scans of individuals considered representative of a generic adult population. The novelty of this method lies in its simplicity so that it does not require any previous training nor a license for its use. The program was developed in scope of introducing an affordable, friendly, semi-automated method of volume quantification of tissues in CT. Utilizing the exported numerical values of the ROIs originally computed by OSIRIX allows the Python algorithm to rely on dependable values. Compared to other available software, VolSEQ also allows for the quantification of tissues to a pixel level, revealing values that would be difficult and even sometimes impossible to identify manually. The interface allows for the selection of the range of HU, so it can also be applied to other targeted tissues for volumetric analysis in CT scans in future studies. Additionally, the novelty of this study was that it quantifies the total volume of the PSIJ and the fat volume within a larger age span (16–82 years) than has been reported in the literature to this date. Its use could be clinically valuable in the detection and quantification of fat content / infiltration in various structures and organs and aid in the potential diagnosis of related conditions.

### Fat appears to be a relevant tissue type defining the ‘healthy’ sacroiliac joint

Fat is observed frequently within the sacroiliac joint ligaments, specifically in and around the interosseous and posterior ligaments^7,28^. It has been hypothesized that fat provides two main functions within the SIJ. Firstly, it allows for the neurovascular structures to travel and supply the syndesmotic SIJ, essentially providing protection to these elements. Secondly, it serves as a mechanical damper within the PSIJ, where it effectively regulates the forces transmitted in this region^28^. As fat is largely incompressible but deformable, compressive forces applied to the posterior pelvis would cause the fat to deform within its given environment. This deformation of fat tissues is limited by the distensibility of the ligamentous meshwork surrounding the fat tissue (typically averaging 15%)^29,30^. To illustrate the morpho-mechanical relationship between fat and ligaments would be to imagine a mattress where the foam compares to the fat and the surrounding fabric compares to the ligamentous tissue^28^.

Previous analyses revealed an age-related correlation with fat volume^7^. This finding was not been replicated in the present study reassessing this research question in a broader age-range. Considering this study has a more suitable age-range for such analyses, one may suggest the current result to be a more realistic reflection of the correlation of fat with age than our previous trials^7^. The lack of correlation showed that age would not be related to fat volumes as previously reported. However, there is a poor correlation between fat volume and total PSIJ volume. Although lower than previously observed, this validates the finding that fat volume is directly proportional to the PSIJ size and is a physiological and functional element of the PSIJ region in general.

### Fat volume seems to remain largely unchanged throughout life and PSIJ volume is sex-dependent in healthy individuals

Analysis of both mean fat and total volume of the PSIJ over time revealed to be similar across the age range. No evidence was found for fat volume decreasing with age therefore, our first hypothesis stating that fat content decreases with age within the SIJ can be rejected. However, it seems that, in general, older individuals tend to retain less fat than the other two younger age groups. As natural degeneration occurs due to age, older individuals may have an overall loss of this force-dampening structural adipose tissue. When separating sexes, it is revealed that there is a trend for an increase in total PSIJ volume with age in females and a decrease over time in males. This could be due to age-related changes that can occur at the SIJ typically involved in osteoarthritis such as ‘joint space narrowing’^31,32^. In this case, normal age-related factors such as osteoporosis, menopause and/or bony fusion of the anterior aspect after cartilage erosion may play a role in the alteration of PSIJ size after 65 years of age. This is sex dependent because of the influence of menopause on cartilage, synovium and bone density^33^. Although difficult to measure because of the use of hormone replacement therapy, these physiological changes brought on by menopause may have a larger impact on the general bony architecture of the SIJ in females. For example, sacral displacement relative to the ilium could be instigated^7^ disturbing the associated fat content within the PSIJ. This clearly differentiates the two sexes on how the PSIJ morphology might evolve in later life.

Furthermore, females have a higher PSIJ volume and total PSIJ fat volume than males. They also have a higher fat percentage as males which suggests that females have more fat locally regardless of their joint size, thus validating our second hypothesis. Considering fat has a load-bearing function it may further compensate for the higher forces and loads subdued by females during life due to their generally smaller articular joint surfaces, but proportionally speaking considering their PSIJ regions are larger, higher fat content is also predictable.

### Fat volume of the PSIJ is side-dependent

Side comparison revealed a higher fat volume on the right side as well as a significant fat percentage difference for the right side. Our third hypothesis suggesting that side has no influence on the fat content within the SIJ must therefore be rejected. Similar results were found previously^7^, and it has been shown that the right leg is often the favored limb regarding side dominance of the lower extremity in tasks such as kicking and jumping for example^34,35^ but generally only 25–45% of the population demonstrate a right leg preference in lower extremity actions^36^. Because of the higher use of one side over the other, we hypothesize that this may influence tendon and ligamentous morphology potentially impacting the ligament-fat ratio within the joint. If the right side is more often the dominant leg, it would typically contain more fat to compensate for the increase in forces and protect neurovascular structures of the joint in response to the increased ligament strain in that area because of the chronic ‘usage’ of the limb. However, the scenario in the scope of this study is arguable as limb dominance is highly task specific^35^ so assuming that the right limb was more dominant than the left in this cohort is a stretch. Chronic limb usage and therefore limb dominance of the patient cohort of this study is unknown, therefore results cannot be reliably assessed and association between fat content in the PSIJ and limb dominance remains to be investigated further.

Limitations of the study include the sample size. Moreover, no pediatric cases were included; therefore, this study is not applicable to individuals younger than 15 years which remains to be studied. In addition, the posterior sacroiliac ligament and the inferior boundary of the articular cartilage were sometimes difficult to determine and, in some instances, had to be separated manually. Thus drawing a line between the ilium and the most posterior part of the sacrum acting as the posterior sacroiliac ligament might bias the annotation process. This approach may have altered the true shape of the structure. Furthermore, the program is not fully independent of manual input and requires the use of OSIRIX or similar software for the creation of the original ROIs. The BMIs (thus, height and weight) of the patients were unknown*;* therefore, it was impossible to consider the impact of BMI on fat volume within the PSIJ. Leg dominance was also unknown for the individuals. Further research should investigate this variable to understand its potential influence on altered loads and stresses on the joint.

## Conclusions

Throughout life, fat volume within the PSIJ remains largely unchanged in asymptomatic healthy and therefore ‘normal’ individuals. The total PSIJ volume is sex-dependent revealing marked differences between males and females specifically after 65 years of age. VolSEQ is a viable and user-friendly method for the quantification of tissue volumes in clinical CT scans that can be applied to clinical casework.

## Data Availability

The data acquired in the course of this study are available from the corresponding author on reasonable request.
